# The Potential Role of Artificial Intelligence in Lung Cancer Screening Using Low-Dose Computed Tomography

**DOI:** 10.3390/diagnostics12102435

**Published:** 2022-10-08

**Authors:** Philippe A. Grenier, Anne Laure Brun, François Mellot

**Affiliations:** 1 Department of Clinical Research and Innovation, Hôpital Foch, 92150 Suresnes, France; 2 Radiology Department, Hôpital Foch, 92150 Suresnes, France

**Keywords:** artificial intelligence (AI), lung cancer screening (LCS), low-dose CT (LDCT), pulmonary nodule, nodule detection, nodule characterization, coronary artery calcifications (CAC), emphysema, quantitative CT, osteoporosis

## Abstract

Two large randomized controlled trials of low-dose CT (LDCT)-based lung cancer screening (LCS) in high-risk smoker populations have shown a reduction in the number of lung cancer deaths in the screening group compared to a control group. Even if various countries are currently considering the implementation of LCS programs, recurring doubts and fears persist about the potentially high false positive rates, cost-effectiveness, and the availability of radiologists for scan interpretation. Artificial intelligence (AI) can potentially increase the efficiency of LCS. The objective of this article is to review the performances of AI algorithms developed for different tasks that make up the interpretation of LCS CT scans, and to estimate how these AI algorithms may be used as a second reader. Despite the reduction in lung cancer mortality due to LCS with LDCT, many smokers die of comorbid smoking-related diseases. The identification of CT features associated with these comorbidities could increase the value of screening with minimal impact on LCS programs. Because these smoking-related conditions are not systematically assessed in current LCS programs, AI can identify individuals with evidence of previously undiagnosed cardiovascular disease, emphysema or osteoporosis and offer an opportunity for treatment and prevention.

## 1. Introduction 

Lung cancer screening (LCS) using low-dose computed tomography (LDCT) has been shown to reduce lung cancer-specific mortality. In 2011, the National Lung Screening Trial (NLST) was the first multicenter randomized controlled trial (over 53,000 current or former smoker participants) to show a 20% decrease in lung cancer deaths after three rounds of annual screening using LDCT and seven years of follow-up, compared to annual screening with chest radiographs [1]. In 2013, the United States Preventive Services Task Force (USPSTF) recommended annual lung cancer screening with LDCT for smokers aged between 55 and 80 years, with at least 30 pack-years of smoking exposure that currently smoke or who have quit smoking within 15 years. Since then, many LDCT-based screening programs have already been implemented in the USA, with the knowledge that the new USPSTF recommendations extended screening to smokers aged 50 to 80 years who have a 20 pack-year smoking history [2,3]. Furthermore, given the success of the UK Lung Cancer Screening trial, implementation programs are currently underway in the UK [4]. In 2020, the results of the Dutch-Belgian NELSON trial, the second largest randomized controlled trial with 15,789 participants, showed a 24% reduction in mortality from lung cancer in a high-risk population of men compared to no screening [5]. The growth-rate assessment for indeterminate nodules was an effective way to reduce the false positive rate to approximately 2%, compared with a 24% false positive rate reported in the NLST [1]. In order to further reduce the false positive rate while maintaining a high sensitivity, various CT reporting systems were developed [6,7,8,9,10,11]. The Lung CT Screening Reporting and Data system (Lung-RADS) developed by the American College of Radiology has been the most used for the reporting of annual screening CT scans in the USA [8]. Such reporting systems can help radiologists to detect, measure, classify and characterize pulmonary nodules, to detect other significant findings, to estimate the malignancy probability of the detected abnormality, and finally propose modalities of the follow-up. The complete analysis of these scans is very challenging and time-consuming, has substantial reader variability, and thus influences the effectiveness of lung cancer screening. One possible solution for this problem is to use artificial intelligence (AI).

Despite the fact that LCS via LDCT scans can reduce the number of deaths from lung cancer, many smokers die of comorbid smoking-related diseases [5,12]. LCS scans contain findings of smoking-related diseases that are not currently systematically assessed. Regan et al. showed that analysis of LCS CT scans extended to these findings allows us to identify individuals with evidence of previously undiagnosed cardiovascular disease, emphysema or osteoporosis that corresponded with adverse events [13]. The identification of those smoking-related comorbidities via LDCT could increase the value of screening with minimal impact on LCS programs. AI solutions could facilitate extended readings of LCS LDCT scans by including assessment of these smoking-related diseases and have a positive impact on the health of many smokers. 

The first objective of this article is to analyze the results of previously published studies that focus on AI solutions, developed specifically either to identify lung nodules or to detect and quantify other smoking-related diseases on chest CT scans. The second objective is to discuss the potential role of these AI solutions to help radiologists in the management of lung nodules and smoking-related diseases on LDCT scans in LCS. 

## 2. Identification of Pulmonary Nodules 

The algorithms developed for pulmonary nodule identification are often referred to as computer-aided detection (CAD) systems. They are designed for different purposes, including lung segmentation, pulmonary nodule detection and classification, and prediction of nodule malignancy.

### 2.1. Lung Segmentation

**Figure 1:**
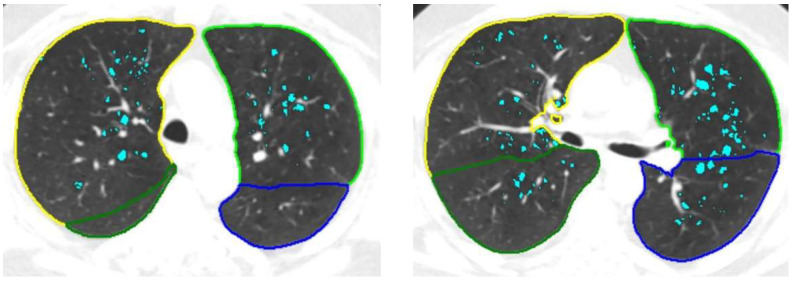

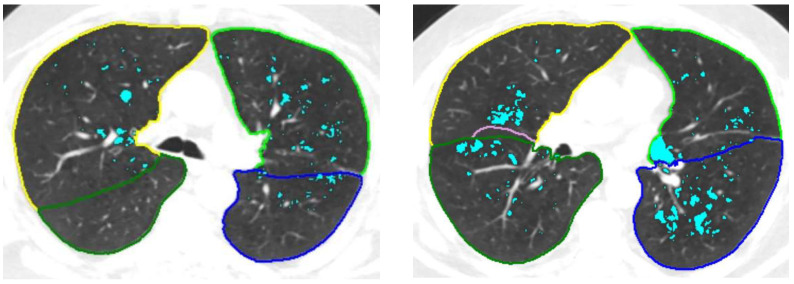
Automatic 3D segmentation of lung and lobe contours and quantitative analysis of emphysema in a 65year-old former smoker using the AI-RAD Companion (Siemens Healthinners) solution. The contours of the left lower lobe are blue and those of the right lower lobe are dark green. The contours of the right upper lobe are yellow and those of the left upper lobe are light green. The voxels that have an attenuation value of less than 950 HU (emphysema) are green turquoise.

Recently, deep learning (DL) algorithms have replaced the classical approaches for automatic lung segmentation. Current state-of-the-art methods use statistical finite element analysis [14], or three-dimensional lung segmentation, improved by deep convolution image-to-image network training, which was successfully implemented by Siemens Healthinners in their AI-RAD Companion framework [15] (Figure 1).

### 2.2. Nodule Detection and Classification

Many articles have been published on AI algorithms for detecting lung nodules [14,16,17,18]. As reported in a review article by Schreuder et al., published in 2021, algorithms demonstrated slightly lower or similar sensitivities compared to radiologists, at the expense of a significant increase in the false positive rate [19]. Even if DL-CAD systems showed a higher detection rate than double readings by radiologists, regardless of nodule size, the false positive rates (per CT scan) of the DL-CAD systems were higher than that of the double readings. Binczyk and associates recently reported the results of new methods to reduce the rate of false positives [14]. Sensitivities of algorithms to detect lung nodule varied from 72.00% to 97.87%, with a rate of false positives varying from 0.42 to 7.90 per case. 

To assess the impact of CAD as a second reader, investigators evaluated the performances of double readings by radiologists and CAD within a subset of 400 patients from the NELSON trial. They showed that 22% of nodules ≥50 mm^3^ were identified solely by CAD, including one instance of lung cancer [20]. In another study, Liang et al. showed that four different CAD systems detected up to 70% of lung cancers not detected by the radiologist, but missed about 20% of the lung cancers that were identified by the radiologist [21]. These results suggest the potential utility of the CAD system in the role of a second reader [22]. 

CAD systems have also been developed to help radiologists to automatically classify nodule types in order to identify the relevant ones. Ciompi et al. developed an AI algorithm for differentiating between the following six nodule types: spiculated, solid, part-solid, non-solid, calcified, and perifissural [23]. The training of the DL system was carried out with data from the Italian MILD screening trial and the validation performed on an independent set of data from the Danish LCS trial, which was also assessed by four independent radiologists. The results showed that the performance of the DL algorithm was within the limits defined by the inter-observer variability in the four experienced readers, thus performing equally to an independent human expert.

**Figure 2:**
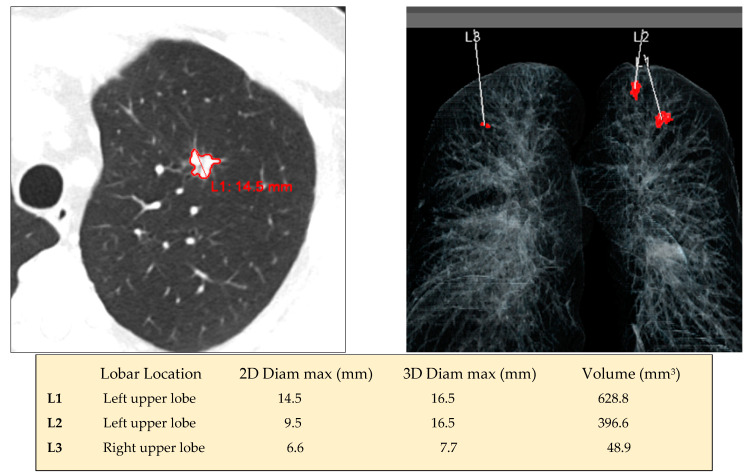
Automatic detection of 3 solid nodules in a 56 current smoker using the AI-RAD Companion (Siemens Healthinners) solution. (**Left top**): axial CT image that shows the results of the automatic segmentation and contouring of the largest left upper lobe nodule. (**Right top**): coronal view that summarizes all lung nodules detected by the CAD system with their location (red). (**Bottom**): table that reports the lobe location, 2D and 3D diameters, and volume of the 3 detected nodules.

With the knowledge that large nodule size is one of the best predictors of malignancy, it can be determined by manually measuring the longest and perpendicular diameters in the transverse plane. Unfortunately, this measurement is prone to inter- and intra-observer variability [24], which can interfere with the diagnostic workup recommendation [25,26]. Volumetric segmentation methods offer the advantage of being less subject to inter- and intra-radiologist variability [27,28]. Considering there is a large variation among different algorithms, the same segmentation algorithm should be used in order to ensure reliable measurement of nodule growth over time. Both 2D and 3D diameters can be automatically obtained from the segmented volume (Figure 2). Using multivariable logistic regression models, Tammemagi et al. showed that both mean diameter and volume measurements of nodule with CAD may provide malignancy risk estimation similar to those of the previously validated PanCan model that was based on radiologist-read LDCT scans and maximum nodule size, with similar predictive abilities between the mean diameter and volume models [29]. Despite many improvements in screening procedures using AI-based solutions to detect and classify pulmonary nodules, the performances of the developed algorithms need to be proven to be quite robust in external datasets before being implemented in routine clinical care.

### 2.3. Malignant Prediction

Accurate estimation of the malignancy risk of pulmonary nodules is essential and remains challenging. In practice, to predict the malignancy of a pulmonary nodule, radiologists currently use the statistical risk model established using patient demographics, nodule size, type and morphology. The most widely used statistical risk model for estimating nodule malignancy risk is the Brock model, also known as the PanCan model [30]. Nevertheless, accurately distinguishing between benign and malignant nodules remains a challenge. 

Several studies [31,32] showed the potential of DL with convolution neural networks (CNNs) in predicting the malignancy risk of a pulmonary nodule through the publicly available Lung Image Database Consortium image collection data set [33]. However, the algorithm performances were evaluated by comparison with the subjective scoring provided by radiologists without a definitive reference standard for malignant and benign nodules. By contrast, Venkadesh et al. used a reference standard based on histopathologic confirmation for malignant lesions and/or follow-up with CT for more than two years for benign nodules [34]. They developed a DL algorithm for malignancy risk estimation of pulmonary nodules by using LDCT scans from the NLST and validated it in the Danish LCS trial (DLCST). The algorithm was based on 2D and 3D CNNs with information from a single CT examination. In the DLCST cohort, the accuracy of the developed algorithm significantly outperformed that of the Pan-Can model. The area under the curve (AUC) was 0.93 vs. 0.90 (*p* = 0.046). At a specificity of 90%, the sensitivities were 84% and 63% for the DL algorithm and the Pan-Can model, respectively. The DL algorithm performances were also assessed on two subsets of cancer-enriched cohorts selected from the DLCST cohort. Both subsets included all lung cancer nodules and twice as many benign nodules. In one subset, the benign nodules were sampled at random, whereas in the second subset, they were size matched to the cancers before sampling at random to remove the effect of nodule size. The DL algorithm performed in a significantly comparable way to thoracic radiologists in both subsets. In addition, it significantly outperformed the Pan-Can model only in the size-matched cancer-enriched subset, indicating that the algorithm takes into account valuable predictive information unrelated to nodule size. One of the limitations of the algorithm was that it considers only one CT examination and not any previous CT images if available; therefore, it is adapted for nodules first observed during screening, similar to the Pan-Can model [35]. On the other hand, for nodules detected during incidence screening, their growth and appearance on the previous CT examinations are important to consider. As nodule growth on CT is the most important predictor of cancer, in order to estimate lung cancer risk, Ardila et al. developed a DL system mainly based on changes in nodule volume [36]. They trained and tested the algorithm on data from 42,290 and 6716 NLST participants, respectively, and validated it retrospectively in an independent clinical dataset of 1139 individuals. In the test set, the model achieved an AUC of 94.4% (95% CI 91.1–97.3%), with a similar result obtained in the external validation set. When multiple scans were available, the model performance was equal to that of radiologists. Huang et al. developed a DL algorithm to identify nodule features that were predictive of malignancy on the screening chest LDCTs within a three-year period [37]. The training set included baseline and follow-up LDCT data from 25,097 NLST participants who had undergone at least 2 LDCT scans. The validation set included LDCT data of 2294 participants from the Pan-Can study. Performance of the AI algorithm score to inform lung cancer incidence was compared with Lung-RADS and volume doubling time. Compared to the Pan-Can model incorporated into Lung-RADS, the algorithm classified a high-risk group that was smaller and had a higher proportion of cancers. Individuals with high AI scores had significantly higher mortality rates compared to those at lower risk. The algorithm also identified more accurately those with very low risks of lung cancer within 2 years. 

Although these studies show promising results, several additional important steps need to be finalized before the AI algorithms can be widely accepted into screening practice. Even if these algorithms appear to demonstrate a potential use to practice LCS, they must undergo multiple iterations of external validation [35]. 

## 3. Assessment of Smoking-Related Diseases (Comorbidities)

Collateral findings of smoking-related diseases are frequently observed on LDCT LCS. Many of them are associated with morbidity and mortality in smokers and are predictive of adverse events. 

### 3.1. Coronary Artery Calcification and Cardiovascular Events

**Figure 3:**
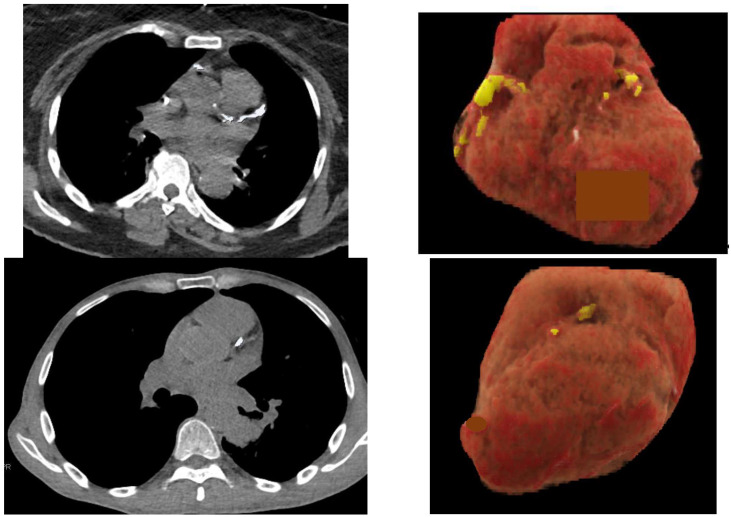
Ungated non contrast chest CT scans in two different smokers with automatic quantification of coronary artery calcifications (CAC) and heart volume using the AI-RAD Companion (Siemens Healthinners) software. Axial CT images show the CAC (**left**) and coronal 3D projection of the heart (**right**) that shows the segmented CAC (yellow). The total CAC and heart volumes were 1175 mm^3^ (category IV) and 620 mL, respectively, in the first subject (**top**), and 187 mm^3^ (category III) and 832 mL, respectively, in the second (**bottom**).

Among the Nelson trial results, after three LDCT scans and ten years of surveillance, the number of deaths from lung cancer (N = 370–21.4%) was similar to the number of deaths from cardiovascular disease (N = 370–21.4%) [5]. Estimates of coronary artery calcifications (CAC) have proved to be a strong predictor of cardiovascular events [38]. Smokers are at risk of adverse events, including myocardial infarctions, strokes and congestive heart failure. Since 2011, we have known that ungated chest CT scans can provide reliable estimations of CAC [39]. The rate of deaths from cardiovascular event increases with the amount of CAC [40,41,42]. So far, a simple semi-quantitative scoring method (none, mild, moderate, or severe) has been used to evaluate CAC on LDCT. When used by cardiac imaging experts, this method globally correlates with conventional CAC score groups. The implementation of AI in a LDCT LCS program can potentially provide a reliable and reproducible numerical value to the calcium score, based on whole heart volume scoring of calcium [43]. This method provides results that closely align with the Agatston scores. The total volume (mm^3^) of calcium within the walls of coronary arteries may be classified on ungated LDCT scans into four categories (<10, 10–100, 101–500, >500 mm^3^) [44]. A score of 4 (>500 mm^3^) is a significant predictor of death from cardiovascular disease (Figure 3) and should lead to further invasive or functional assessment examinations (stress testing, echocardiography, coronary angiography). Furthermore, guidelines recommend that lipid lowering medication should be given to patients with increased CAC [45].

In 2020, Lee et al. summarized the current AI-based applications for CAC scoring and their potential clinical impact [46]. Recent developments of DL-based algorithms have provided considerable progress in CAC evaluation. Several investigations that evaluated the clinical role of DL solutions in CAC assessment showed excellent agreement between those algorithms and manual scoring [46,47]. More specifically, Chao et al. trained a DL cardiovascular disease risk prediction model with 30,286 LDCT scans from the NLST [48]. They achieved an AUC of 0.871 on a separate test set of 2085 subjects and identified patients with high cardiovascular mortality risks (AUC of 0.768). Then, the same investigators validated their model against ECG-gated cardiac CT-based markers obtained from an independent dataset of 335 subjects. Hence, the potential of obtaining a quantitative and reliable cardiovascular disease risk score by analyzing the same scans as those for LCS may benefit a large patient population. In addition, the automatic exclusion of LDCT scans with a negative test for CAC could significantly reduce the workload of radiologists [49]. 

### 3.2. Emphysema 

To detect the presence of emphysema and assess its extent in smokers make sense for several reasons. 1- Centrilobular emphysema is associated with a greater risk of lung cancer, which also increases with emphysema severity [50], and as a result, its identification provides important information for the malignancy risk estimation. 2- Smokers with normal spirometry may have emphysema that is visible on CT scans [51]. 3- Undiagnosed COPD is common among smokers and is associated with exacerbation-like respiratory events [52]. 4- The presence of emphysema on a chest CT scan in smokers with or without symptoms, but with normal spirometry, has proven to be predictive of emphysema progression, lung function loss, and increased risk of mortality [53]. 5- The presence and extent of emphysema visually assessed on chest CT scans are both associated with an increased risk of mortality [54]. 

DL-based solutions have been developed to detect and quantify emphysema on LDCT examinations [55,56,57] (Figure 1). In practice, lung abnormalities are visually assessed using high-contrast thin-slice images reconstructed from raw scan data using sharp kernels, despite increased image noise. On the opposite side, accurate CT quantification requires low-contrast thin-slice images with low noise, which are reconstructed with soft kernels. Investigators have applied DL techniques for converting sharp-kernel images to soft-kernel-like images and normalizing CT kernels effects in order to reduce the kernel-induced variability in lung density measurements [56,57]. Hence, the DL algorithm has the potential to increase the accuracy of emphysema quantification, and can allow reliable surveillance of emphysema in LCS, even if follow-up CT scans are acquired with different reconstruction kernels. To demonstrate that emphysema quantification was feasible on LDCT scans using DL-based conversion strategies, the Korean Society of Imaging Informatics in Medicine organized a challenge between 24 November 2020 and 26 January 2021 [58]. Using the training set, each of the seven participating teams developed an algorithm that provided converted LDCT by changing the pixel values of LDCT to simulate those of standard-dose CT. 

### 3.3. Osteoporosis and Fragility Fractures 

**Figure 4:**
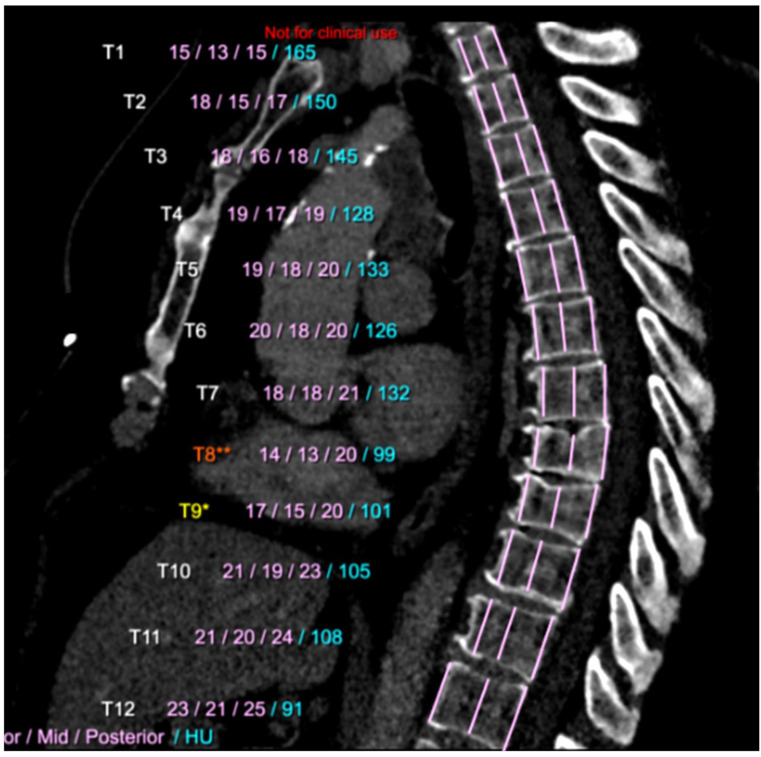
Lateral reconstruction of LDCT images in a COPD patient. Labelling of thoracic vertebra and measures of vertebral bodies heights were automatically obtained through the AI-RAD Companion (Siemens Healthinners, Erlangen, Germany) software. The heights of the T8 and T9 vertebral bodies were lower than those above and below. The bone attenuation values measured within the T8 and T9 vertebral bodies were also lower than those above and below (osteoporosis and minimal collapse of the T8 vertebral body).

Osteoporosis and fragility fractures that occur in aging adults are associated with death, loss of independence and decline in physical functionality [59,60]. Osteoporosis itself is associated with mortality and morbidity in the elderly, especially among patients with COPD [61,62]. Osteoporosis is highly prevalent in COPD patients and bone attenuation has been shown to be lower in COPD subjects, compared with smoker and nonsmoker controls [63]. Bone attenuation measured on routine chest CT has proved to correlate strongly with bone marrow density assessed on DXA in patients with COPD [64]. Ohara et al. showed that in COPD patients, the extent of pulmonary emphysema was significantly correlated with decreased bone density [65]. In addition, van Dort and associates evaluated bone attenuation in vertebrae T_4_–T_12_ and prevalent and incident vertebra fractures in 1239 individuals included in the ECLIPSE study, with baseline and 1-year and 3-year follow-up CT scans [66]. The results demonstrated that in former smokers with and without COPD, the combination of bone attenuation and prevalent vertebra fractures was strongly associated with the short-term risk of incident vertebra fractures. In 2021, Fang et al. developed a DL-based algorithm, allowing fully automatic segmentation of vertebral body and bone mineral density calculation in CT images. They demonstrated that a DL algorithm could automatically identify osteoporosis, osteopenia, and normal bone mineral density in CT images [67]. At the same time, Pan et al. developed a DL-based system to automatically measure bone mineral density on LDCT scans obtained for LCS, with the objective to screen opportunistic osteoporosis [68]. They trained and tested the DL model with 200 annotated LDCT scans to segment and label all vertebral bodies. Then, the mean CT numbers of the trabecular area of target vertebral bodies were obtained based on the segmentation mask, and mapped with their bone mineral densities collected from approved software used for osteoporosis diagnosis. The authors evaluated the diagnostic performance of the developed system using an independent dataset of 374 LDCT scans with standard bone mineral densities and osteoporosis diagnosis. The DL algorithm achieved 86.6% and 97.5% accuracies for vertebral body segmentation and labeling, respectively. Linear regression and Bland–Altman analyses showed good agreement between the predicted bone mineral measure and the ground truth, with correlation coefficients of 0.964–0.968 and mean errors of 2.2–4.0 mg/cm^3^. The sensitivity and specificity of the developed system for detecting osteoporosis were 85.7% and 99.7%, respectively, with an AUC of 0.927. These diagnostic performances seem to be quite promising for automatic measurement of vertebral bone mineral density in opportunistic osteoporosis screening, using LDCT scans obtained for LCS (Figure 4).

## 4. Simultaneous Assessment of Lung Nodule, CAC, Emphysema and Osteoporosis

Simultaneous identification of lung nodule and smoking-related diseases on LDCT scans may be obtained using AI solutions that combine the simultaneous processing of several algorithms. Using an AI CNN prototype (AI-RAD Companion, Siemens Healthineers) that automatically detects pulmonary nodules and quantifies CAC volume on chest LDCT, Chamberlin et al. compared results to expert radiologists in a retrospective cohort of 117 patients who underwent LDCT [69]. All subjects were used for lung nodule analysis, and 96 subjects met the criteria for CAC volume analysis. Agreement of the AI findings with experts was excellent, with high sensitivity and specificity. The authors also found that the AI-based algorithm results correlated with adverse outcomes at the 1-year follow-up and showed improvement of the prediction of major cardiopulmonary outcomes, including major adverse cardiac events and lung cancer. 

Currently, the AI-RAD Companion solution includes several DL-algorithms that provide simultaneous automatic results, which are as follows: 1- segmentation of the lungs and lobes (Figure 1), 2- detection and quantification of emphysema (volume percentage per lobe and per lung) (Figure 1), 3- detection of solid nodules that measure 6 mm or more using diameter and volume measurements (Figure 2), 4- segmentation and volume quantification of coronary artery calcifications (Figure 3), 5- measures of aortic diameters that are strictly perpendicular to the central axis of the vessel at different segments of the thoracic aorta, 6- labeling of thoracic vertebra bodies with measurements of vertebra bodies’ heights and bone attenuation (Figure 4). In a few minutes time, the results are available and automatically implemented in the PACS. This complete solution is particularly well adapted for a platform that allows personalized risk estimation of cardiovascular and respiratory adverse events, and bone fracture, combined with a LCS program using LDCT. 

## 5. Conclusions

Extensive efforts have been made to develop AI solutions for pulmonary nodule detection, classification, and malignancy estimation on chest CT scans. The most recent algorithms developed with DL methods have similar performance or are close to double reading by radiologists. After deep external validation made of multiple iterations, these algorithms could be used as a second reader in LCS programs. At the same time, DL algorithms have been developed to detect and quantify on chest CT scans other smoking-related diseases, including CAC, emphysema, and osteoporosis, that have an impact on morbidity and mortality. 

Despite the absence of official recommendations about the role of AI applications for LCS, these new achievements should be kept in mind when developing new LCS programs. AI algorithms that provide a second reading for lung nodule detection and simultaneous assessment of smoking-related comorbidities could increase radiologist confidence, shorten turnaround time, provide better patient outcomes, and eventually reduce costs by improving disease prevention in this high-risk population. 

## Data Availability

Not applicable.
